# Supplementary material for: A Small Molecule That Promotes Cellular Senescence Prevents Fibrogenesis and Tumorigenesis
Source: Int J Mol Sci. 2022 Jun 20;23(12):6852. doi: 10.3390/ijms23126852 (PMC9224374; doi:10.3390/ijms23126852)
Supplement: Supplementary file 1 [file ijms-23-06852-s001.zip › ijms-1762033-supplementary.pdf]

GeneName	Desc	log2fc	p-value	q-value
**NRP1**	neuropilin 1	-2.78	5.00E-05	0.0159
**MMP1**	matrix metallopeptidase 1 (interstitial collagenase)	-3.12	5.00E-05	0.0159
**FARP1**	FERM, RhoGEF (ARHGEF) and pleckstrin domain protein 1 (chondrocyte-derived)	-2.53	5.00E-05	0.0159
**THSD4**	thrombospondin, type I, domain containing 4	-4.56	5.00E-05	0.0159
**NPTX1**	neuronal pentraxin I	-3.84	5.00E-05	0.0159
**SLC14A1**	solute carrier family 14 (urea transporter), member 1 (Kidd blood group)	-4.74	5.00E-05	0.0159
**CCBE1**	collagen and calcium binding EGF domains 1	-3.14	5.00E-05	0.0159
**SPPL2B**	signal peptide peptidase like 2B		5.00E-05	0.0159
**TBC1D5**	TBC1 domain family, member 5	-3.66	5.00E-05	0.0159
**ABI3BP**	ABI family, member 3 (NESH) binding protein	-2.92	5.00E-05	0.0159
**PTX3**	pentraxin 3, long	-4.05	5.00E-05	0.0159
**LPP**	LIM domain containing preferred translocation partner in lipoma	-3.72	5.00E-05	0.0159
**SCFD2**	sec1 family domain containing 2	-3.73	5.00E-05	0.0159
**MAPK10**	mitogen-activated protein kinase 10	-3.12	5.00E-05	0.0159
**CAMK2D**	calcium/calmodulin-dependent protein kinase II delta	-3.02	5.00E-05	0.0159
**RPS6KA2**	ribosomal protein S6 kinase, 90kDa, polypeptide 2	-2.77	5.00E-05	0.0159
**ANLN**	anillin, actin binding protein	-2.75	5.00E-05	0.0159
**CACNA2D1**	calcium channel, voltage-dependent, alpha 2/delta subunit 1	-3.13	5.00E-05	0.0159
**EXOC4**	exocyst complex component 4	-3.14	5.00E-05	0.0159
**TNFRSF11B**	tumor necrosis factor receptor superfamily, member 11b	-2.72	5.00E-05	0.0159
**PRUNE2**	prune homolog 2 (Drosophila)	-2.54	5.00E-05	0.0159

Supple. Table S1

Metabolic shift / Histone modification

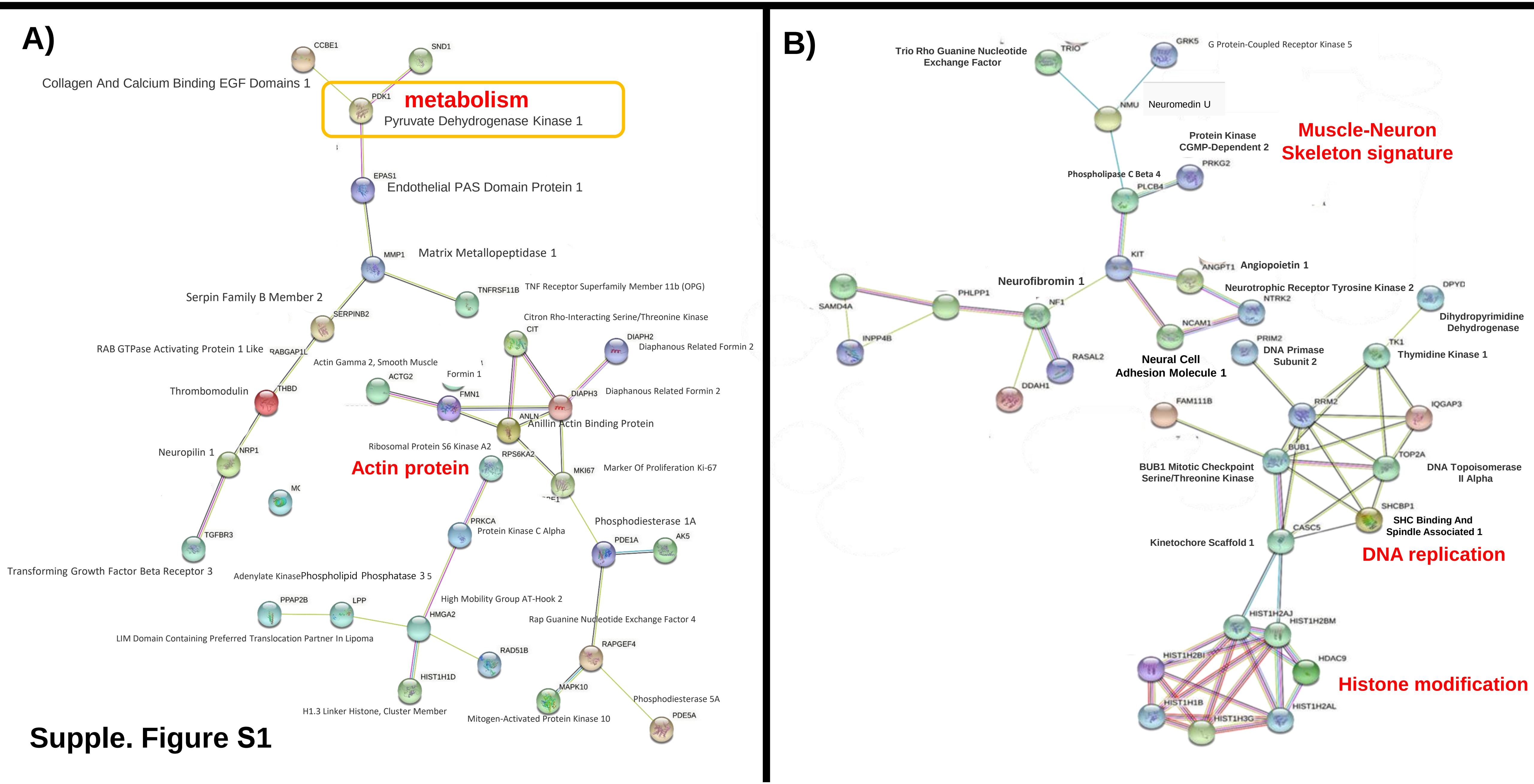

Supple. Figure S1

DNA replication / Chromatin Remodeling / Cell Division Control

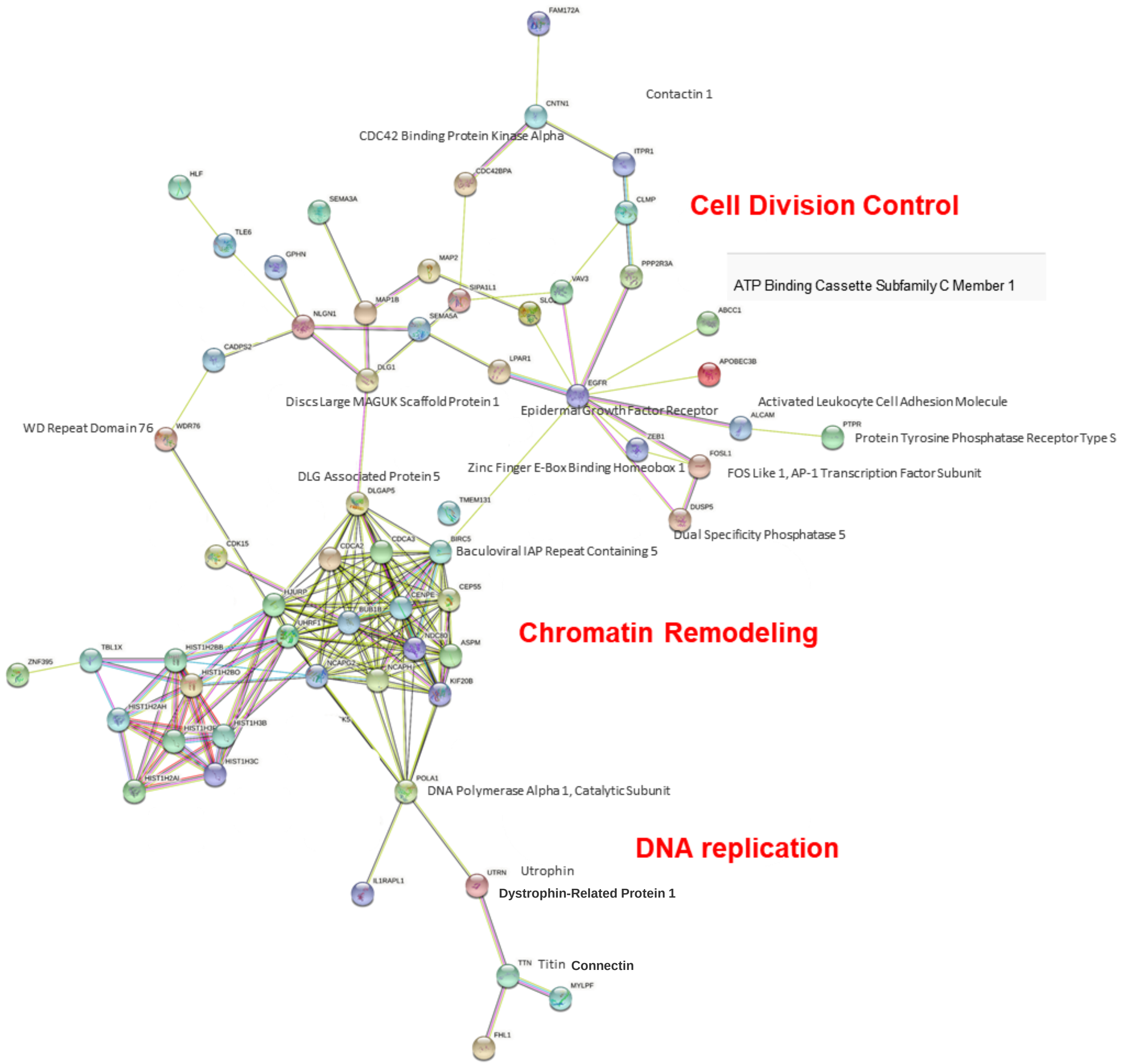

Supple. Figure S2

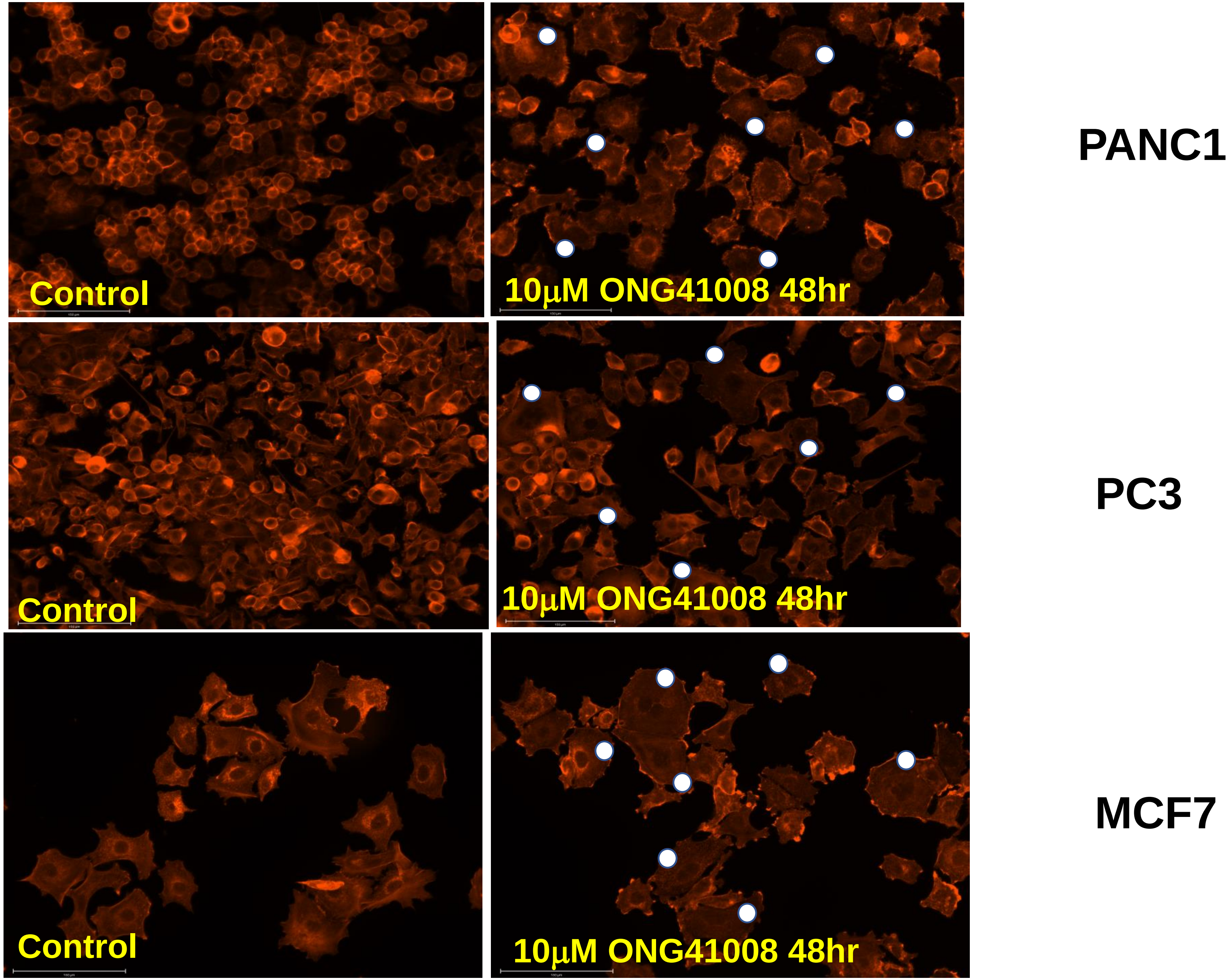

Supple. Figure S3

A) A549(p53) 24h

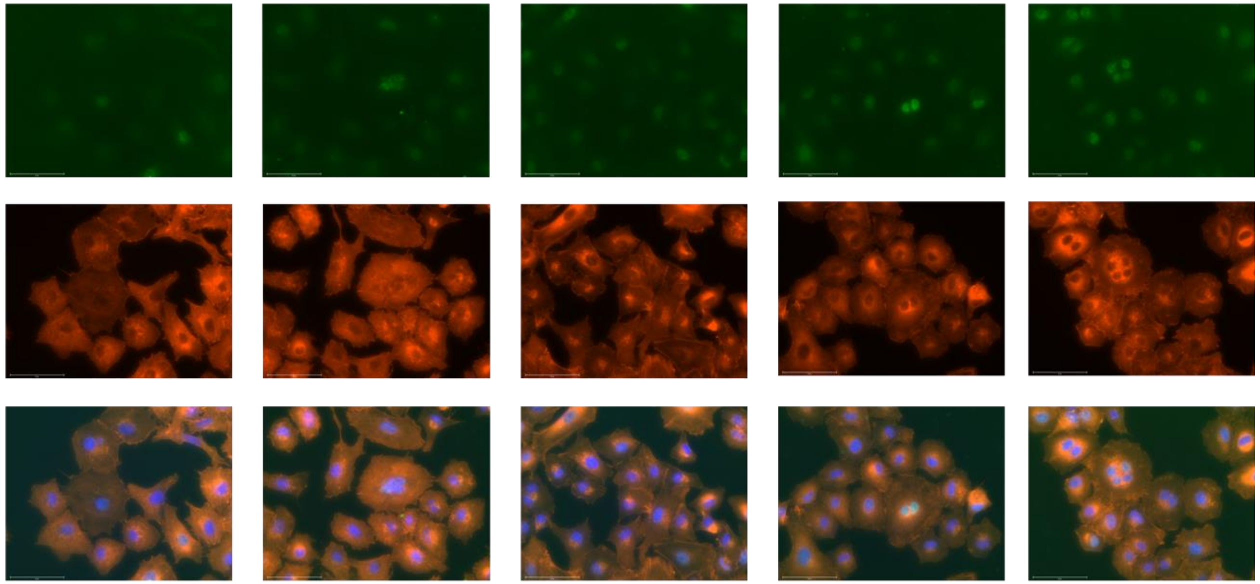

B) A549(p21) 24h

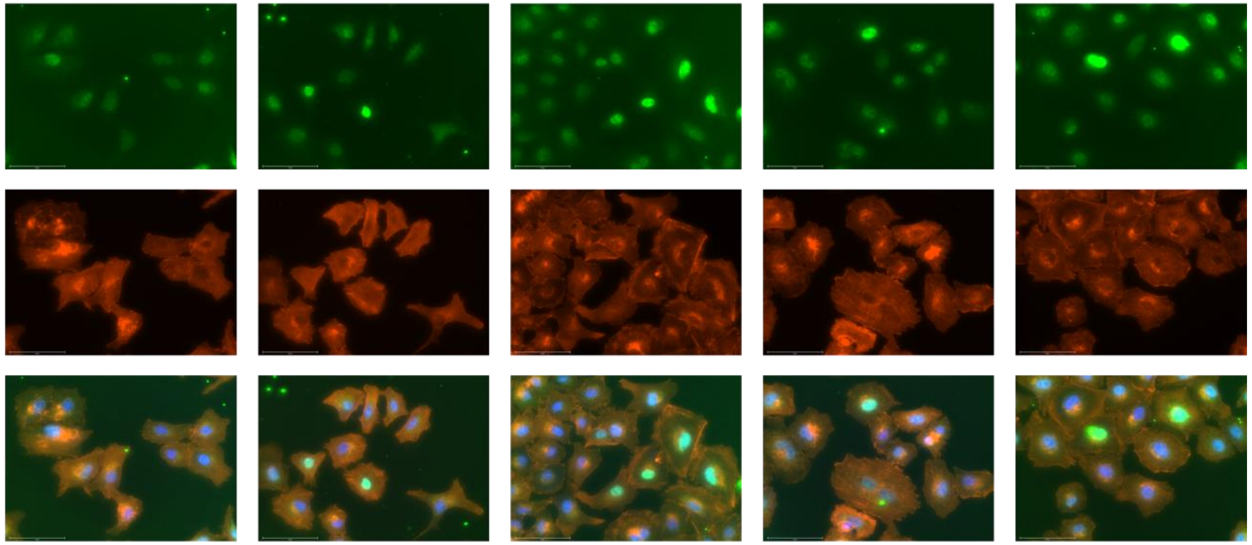

C) A549(p16) 24h

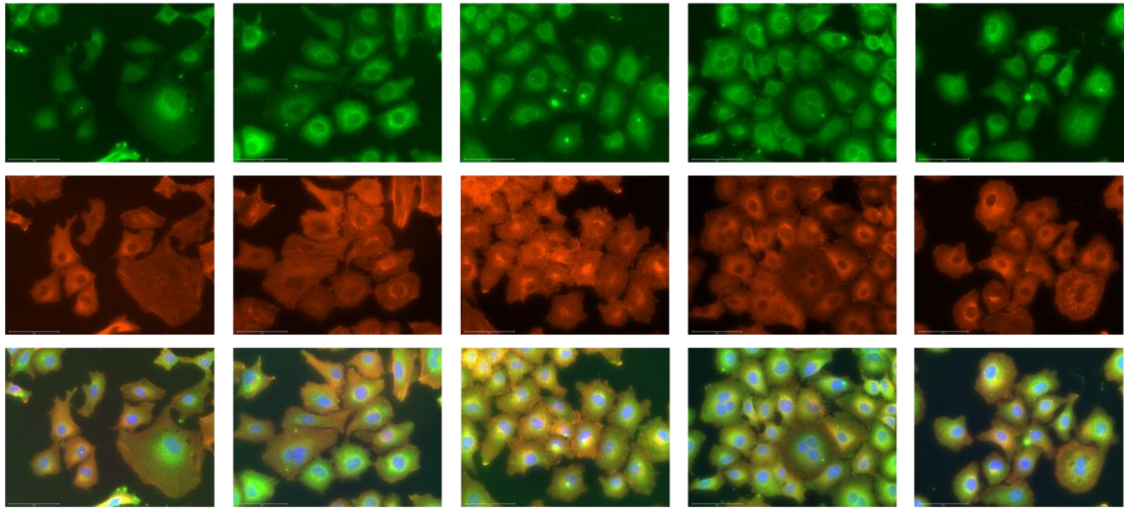

Supple. Figure S4

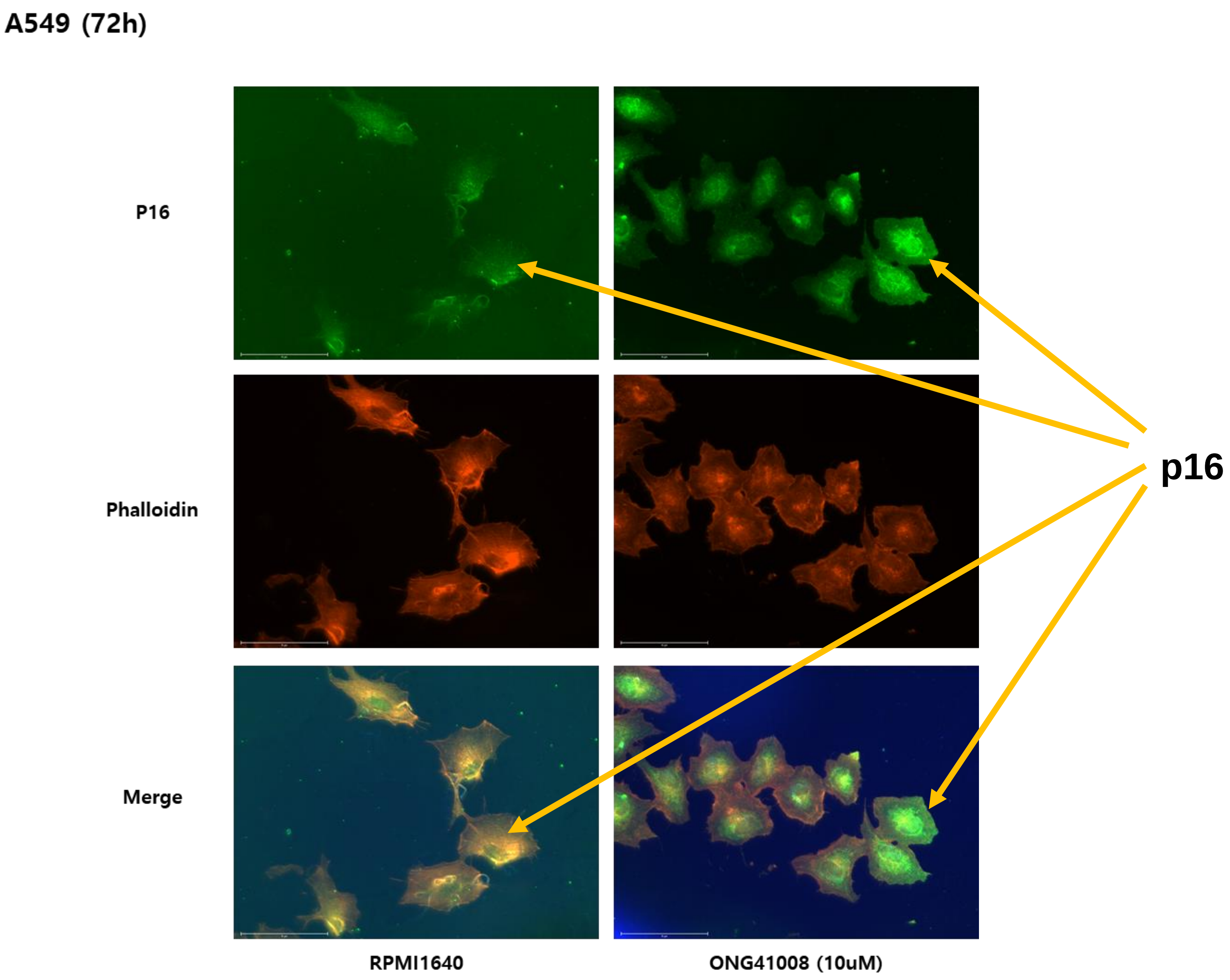

Supple. Figure S5

A)

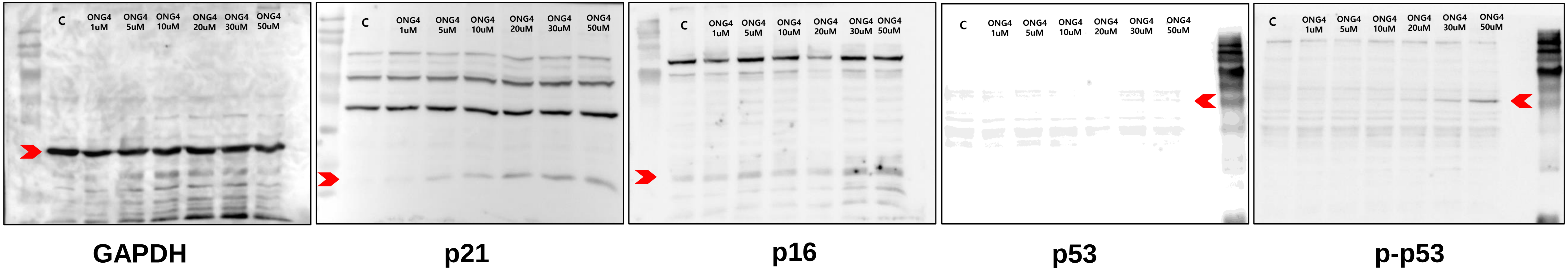

B)

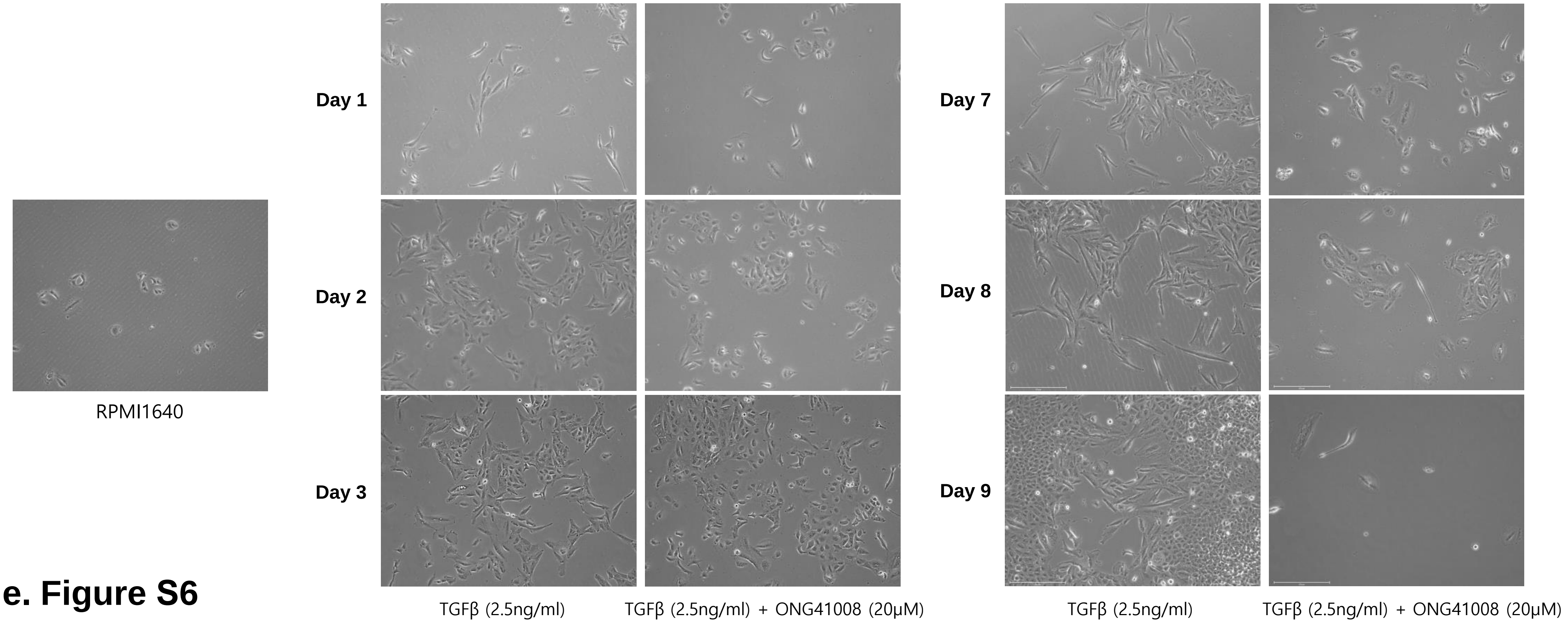

Supple. Figure S6
